# Preparation of (*S*)-1-Halo-2-octanols Using Ionic Liquids and Biocatalysts †

**DOI:** 10.3390/molecules14104275

**Published:** 2009-10-23

**Authors:** Mireia Oromí-Farrús, Jordi Eras, Núria Sala, Mercè Torres, Ramon Canela

**Affiliations:** 1 Chemistry Department, Lleida University, 25198-Lleida, Spain; E-Mails: mireia.oromi@quimica.udl.cat (M.O-F.); eras@quimica.udl.cat (J.E.); 2 Food Technology Department, Lleida University, 25198-Lleida, Spain; E-Mail: nsala@tecal.udl.cat (N.S.)

**Keywords:** (*S*)-1-bromomethyl-1-heptanol, (*S*)-1-chloromethyl-1-heptanol, lipases, enzymatic resolution

## Abstract

Preparation of (*S*)-1-chloro-2-octanol and (*S*)-1-bromo-2-octanol was carried out by the enzymatic hydrolysis of halohydrin palmitates using biocatalysts. Halohydrin palmitates were prepared by various methods from palmitic acid and 1,2-octanediol. A tandem hydrolysis was carried out using lipases from *Candida antarctica* (Novozym^®^ 435^)^, *Rhizomucor miehei* (Lipozyme IM), and “resting cells” from a *Rhizopus oryzae* strain that was not mycotoxigenic. The influence of the enzyme and the reaction medium on the selective hydrolysis of isomeric mixtures of halohydrin esters is described. Novozym^®^ 435 allowed preparation of (*S*)-1-chloro-2-octanol and (*S*)-1-bromo-2-octanol after 1–3 h of reaction at 40 °C in [BMIM][PF_6_].

## 1. Introduction

Racemic halohydrins and haloesters can be obtained using diverse approaches [1]. Among them, our research group has developed a direct methodology for halohydrin ester formation using ionic liquids (ILs) and potassium halides [2]. These compounds can be stereoselectively hydrolyzed using an enzymatic approach [3,4]. Enantiomerically pure halohydrins are useful intermediates in the preparation of agrochemicals, pharmaceuticals, and liquid crystals [5,6].

Enzymes can catalyze a variety of reactions with high specificity and under mild conditions such as low temperature and atmospheric pressure; biotransformations are looked on as a way of resolving environmental problems involved in chemical procedures [7,8,9,10]. Furthermore, ILs have a very low vapor pressure and are considered a greener alternative to volatile organic solvents. Their solvent properties may be easily controlled by altering the nature of their cations and anions [11]. Although the application of ILs as a solvent in biocatalysis is very recent and only three articles had been published up to 2002 [12,13,14], many enzymatic reactions and their applications have been described since then [15,16,17]. Combining both technologies allows the design of an environmentally friendly process.

The aim of this work was to evaluate the suitability of commercial lipases and resting cells prepared from a nonmycotoxigenic strain of *Rhizopus oryzae* in the regio- and stereoselective hydrolysis of halohydrin esters for preparing (*S*)-1-bromo-2-octanol and (*S*)-1-chloro-2-octanol. These compounds have been used for the synthesis of epoxides [18], tetrathiafulvalene derivatives [19] and higher-molecular-weight aliphatic sulfonic acids [20]. The compound ONO-2506, an agent that suppresses astrocyte activation [6], and some adenosine deaminase inhibitors [21] have also been synthesized from these halohydrins.

## 2. Results and Discussion

In a previous paper, we described a chemoenzymatic method for preparing (*R*)-4-chloro-2-butanol from 1,3-butanediol [3]. First, the synthesis of isomeric mixtures of chlorohydrin esters was carried out using this diol and several alkyl carboxylic acids. The isomeric mixtures were then hydrolyzed selectively using both fungal resting cells and commercial lipases in a tandem enzymatic system. The regioselectivity and enantioselectivity of these hydrolyses depended on the solvent, the temperature, the acyl alkyl chain, and the biocatalyst used. The best results were obtained using *t*-butanol as solvent and C_16_ and C_18_ carboxylic acids, and by carrying out the hydrolytic reactions at 40 °C. Considering these previous results and our interest in preparing enantiomers of 1-halo-2-octanols, a set of experiments was conducted to study the extension of the described method to 1-halo-2-octyl palmitates prepared according to the methods already described. Using these methods, isomeric mixtures of 1-chloro-2-octyl (**1a**) and 2-chloro-1-octyl (**2a**) palmitates (regioisomeric ratio 90:10) and 1-bromo-2-octyl (**1b**) and 2-bromo-1-octyl (**2b**) palmitates (regioisomeric ratio 75:25) can be obtained. The tandem enzymatic reaction was then studied comparing *t*-butanol, the best organic solvent found in our previous study, with some ILs.

### 2.1. Influence of Solvents and Biocatalysts on the Regioselectivity of the Process

Table 1 shows the influence of the solvents on the regioselectivity of various biocatalysts in the presence of an isomeric mixture of chlorooctyl (**1a, 2a**) and bromooctyl (**1b, 2b**) palmitates. The hydrolyses were performed at 40 °C for 24 h unless otherwise indicated in the Table. 

The best results were obtained for Lipozyme. Although *t*-butanol and [OMIM][BF_4_] gave similar regioselectivity for the chlorooctyl palmitates (**1a, 2a**), 24 h of reaction in *t*-butanol was enough to obtain 100% hydrolysis of **2a**. However, the regioselectivity of Lipozyme for the hydrolysis of bromooctyl palmitates (**1b, 2b**) was much better in [OMIM][BF_4_] than in *t*-butanol. These results differ from those obtained with chlorobutyl esters, where resting cells of *R. oryzae* and *t*-butanol had been shown to be the best combination for the regioisomeric hydrolysis of the corresponding esters [3]. Moreover, *t*-butanol is also a nonideal solvent for regioisomeric hydrolysis of bromooctyl palmitates with any of the biocatalysts studied, showing that the introduction of a more bulky halogen group than chloro dramatically diminishes the enzyme regioselectivity in this solvent. Although [BMIM][PF_6_] has only been tested using Novozym, a considerable increase in the hydrolytic activity for the four isomers was observed. These results prompted us not to test this IL with the other biocatalysts.

### 2.2. Influence of Solvents and Biocatalysts on the Enantioselectivity of the Process

Table 2 shows the influence of the solvents on the enantioselectivity of various biocatalysts in the presence of an enantiomeric mixture of 1-chloro-2-octyl (**1a**) and 1-bromo-2-octyl (**2a**) palmitates. The hydrolysis was performed at 40 °C for 24 h unless another time is indicated in the Table. The biocatalysts and solvents showing hydrolysis above 30% of 1-halo-2-octyl regioisomers (**1a** and **2a**) in the previous experiments were the only ones studied in this case. In all cases, the (*S*)-isomers were hydrolyzed more quickly than the corresponding (*R*)-isomers. These results were the opposite of those observed previously in the hydrolysis of chlorobutyl esters [3], but in accordance with the results described by Rotticci *et al.* [4] for similar esters of the same haloalcohols. The best results were obtained for Novozym^®^ 435 and [BMIM][PF_6_] in terms of *ee* and E values. Both values are better than the values described previously for similar approaches. In contrast, reduction of α−haloketones with recombinant whole cells bearing an alcohol dehydrogenase and a glucose dehydrogenase [22], and dehalogenation of haloalcohols using dehalogenases gave the corresponding haloalcohols with similar *ee* [23]. Thus, kinetic resolution of 1-chloro-2-octanol and 1-bromo-2-octanol with *Candida antarctica* lipase B and hexane gave the two (*S*)-alcohols with an *ee*/E of 41%/14 and 56%/7.6 respectively [4]. Moreover, kinetic resolution of 1-chloro-2-octanol using PS-C “Amano” II and toluene produced the (*R*)-isomer with an *ee*/E of only 28%/3 [23].

Using [BMIM][PF_6_] we obtained *ee_s_*/E values higher than 98%/340 in both cases (Table 2). Moreover, the reaction can be finished in 3 h using [BMIM][PF_6_]. If the reaction time was increased, the percentage of hydrolysis increased but the percentage *ee_s_* of the reaction product decreased (data not shown). Several publications have already shown that using this IL and *C. antarctica* lipases for the resolution of enantiomeric alcohols by esterifications [24,25], transesterifications [26,27], and alcoholysis reactions [13] gave better results than using conventional organic solvents.

## 3. Experimental 

### 3.1. General Procedures

Reactions were performed in 3 ml Eppendorf vials by shaking the mixture at 1,400 rpm and 40 °C. ^1^H- and ^13^C-NMR spectra were recorded on a Varian Unity 400 spectrometer (at 400 MHz for ^1^H-NMR and at 100 MHz for ^13^C-NMR) using CDCl_3_ as solvent, as indicated. Chemical shifts are reported in ppm (δ) relative to the TMS signal. Chiral gas chromatography analyses were carried out with a capillary column CP Chirasil–DEX CB (Varian) (25 m × 0.25 mm diameter, 0.25 μm film thickness). Hydrogen (2.8 mL/min) was used as the carrier gas. *T*_injector_ = 230 °C, *T*_detector_ = 250 °C. The GC temperature was programmed at 60 °C and held for 30 min, ramped at 10 °C/min to 180 °C and held for 2 min. Flash column chromatography (FCC) and analytical thin-layer chromatography (TLC) were performed using silica gel 230–400 mesh and precoated silica gel 60 F254 Merck plates, respectively. 1,2-Octanediol was obtained from Acros Organics. (*R*)-(+)-1,2-octanediol and immobilized lipase from *Mucor miehei* (Lipozyme^®^) were purchased from Sigma-Aldrich. [BMIM][PF_6_] was obtained from Merck. Palmitic acid was obtained from Riedel-de Haën. Immobilized lipase from *Candida antarctica* (Novozym^®^ 435) was a gift from Novo Nordisk. Resting cells of *Rhizopus oryzae* were obtained by the method described in Méndez *et al* [3]. [OMIM][PF_4_] was synthesized at Imperial College, London, U.K. All chemicals were used without further purification.

### 3.2. Procedure for the Syntheses of Halohydrin Palmitates

*1-Chloro-2-octyl palmitate* (**1a**) *and 2-chloro-1-octyl palmitate regioisomer* (**2a**): The title compounds were obtained in 85% overall yield as a 90:10 regioisomeric mixture using the method described in Eras *et al.* [29].

*1-Bromo-2-octyl palmitate* (**1b**) *and 2-bromo-1-octyl palmitate regioisomer* (**2b**): The title compounds were obtained in 80% overall yield as a 75:25 regioisomeric mixture using the method described in Oromí-Farrús *et al.* [2].

### 3.3. Procedure for the Syntheses of Halohydrin Standards

(*R*)-(+)-1,2-octanediol was used to obtain the corresponding haloesters as described above. The synthesis of the corresponding halohydrin mixtures was carried out by reduction of the parent esters as described in Wilen *et al.* [30]. The absolute configuration was assigned assuming an S_N_2 process for the synthesis of the corresponding halohydrin esters [15]. These haloalcohols were used as external standards in the chiral chromatographic analyses.

### 3.4. General Procedure for Optimizing the Enzymatic Hydrolysis of Halohydrin Esters

Hydrolysis in *t*-butanol. Thirty milligrams of either lyophilized mycelium or commercial enzyme, water (5 μL) and a 0.17 M solution (0.5 mL) of each halohydrin palmitate in *t*-butanol were added to each vial. The reaction mixtures were incubated as described above for 24 h. Final mixtures were centrifuged at 3,000 rpm for 2 min and the clear supernatant was recovered by decantation. The organic solvent was evaporated with a nitrogen stream and halohydrin esters and haloalcohols were quantified by ^1^H-NMR using 1,4-dichlorobenzene as the internal standard. Chiral GC-FID was used to determine the corresponding enantioselectivity of the process. All reactions were carried out in duplicate.

Hydrolysis in ionic liquids. One-half milliliter of a 0.17 M solution of each halohydrin palmitate in dichloromethane was added to each vial. The solvent was evaporated with a nitrogen stream, and 30 mg of either lyophilized mycelium or commercial enzyme, water (5 μL) and a solution of each IL (0.4 g) were added to each vial. The reaction mixtures were incubated as described above for 24 h or 3 h, depending on the ionic liquid and biocatalyst (see Table 1 and Table 2). Final mixtures were extracted with hexane (4 × 0.4 mL). The hexane extracts were joined together and evaporated with a nitrogen stream. Halohydrin esters and haloalcohols were quantified by ^1^H-NMR using 1,4-dichlorobenzene as the internal standard. Chiral GC-FID was used to determine the corresponding enantioselectivity of the process. All reactions were carried out in duplicate.

### 3.5. Procedure for Preparing (S)-2-Chloro-1-octanol *(**4a**)*

A mixture of 1-chloro-2-octyl palmitate (**1a**) and 2-chloro-1-octyl palmitate regioisomer (**2a**) (685,2 mg, 1,7 mmol), prepared as described above, Lipozyme (600 mg), water (100 μL) and *t*-butanol (10 mL) were distributed among 20 vials. The reaction mixtures were stirred at 40 °C for 24 h, combined, quenched with saturated sodium bicarbonate solution (10 mL) and then extracted with *t*-butyl methyl ether (15 mL). The organic extract was washed with water (15 mL), dried over MgSO_4_ anhydrous and concentrated *in vacuo*. The crude reaction mixture was purified using FCC to give 479.6 mg (1.2 mmol) of 1-chloro-2-octyl palmitate (**1a**). Two hundred seventy-four milligrams (0.7 mmol) of this ester, 240 mg of Novozym^®^ 435, 40 μL of water, and 3.2 g of [BMIM][PF_6_] were distributed among 8 vials. The reaction mixtures were stirred at 40 °C for 3 h, joined together, quenched with saturated sodium bicarbonate solution, and then extracted with hexane. The organic extract was dried over MgSO_4_ anhydrous and concentrated in vacuo. The crude reaction mixture was purified using FCC to give 50 mg (0.3 mmol) of (*S*)-1-chloro-2-octanol (**4a**). The chloroalcohol was identified by NMR and chiral gas chromatography. ^1^H-NMR δ ppm 0.83 (d, *J* = 7.03 Hz, 3H) 1.13–1.32 (m, 7H) 1.40–1.51 (m, 1H) 1.51–1.71 (m, 2H) 2.05 (d, *J* = 4.64 Hz, 1H) 3.41 (dd, *J* = 11.33, 7.03 Hz, 1H) 3.58 (dd, *J* = 11.33, 3.13 Hz, 1H) 3.67–3.78 (m, 1H); ^13^C-NMR δ ppm 14.28 (2C, CH_3_) 22.79 (1C, CH_2_) 25.34 (1C, CH_2_) 29.77 (1C, CH_2_) 31.92 (1C, CH_2_) 34.30 (1C, CH_2_) 49.29 (1C, CH_2_) 74.01 (1C, CH) [31]; chiral GC R_t_: 10.33 min.

### 3.6. Procedure for Preparing (S)-2-Bromo-1-octanol *(**4b**)*

A mixture of 1-bromo-2-octyl palmitate (**1b**) and 2-bromo-1-octyl palmitate regioisomer (**2b**) (760.8 mg, 1.7 mmol), prepared as described above, Lipozyme (600 mg), water (100 μL) and [OMIM][BF_4_] (8 g) were distributed among 20 vials. The reaction mixtures were stirred at 40 °C for 24 h, combined, quenched with saturated sodium bicarbonate solution (10 mL) and then extracted with hexane (20 mL). The organic extract was washed with water (20 mL), dried over MgSO_4_ anhydrous and concentrated *in vacuo*. The crude reaction mixture was purified using FCC to give 485.0 mg (0.6 mmol) of 1-bromo-2-octyl palmitate (**1b**). Three hundred and four milligrams (0.7 mmol) of this ester, Novozym^®^ 435 (240 mg), water (40 μL) and [BMIM][PF_6_] (3.2 g) were distributed among eight vials. The reaction mixtures were stirred at 40 °C for 3 h, combined, quenched with saturated sodium bicarbonate solution (7 mL) and then extracted with hexane (15 mL). The organic extract was dried over MgSO_4_ anhydrous and concentrated *in vacuo*. The crude reaction mixture was purified using FCC to give 42 mg (0.2 mmol) of (*S*)-1-bromo-2-octanol (**4b**). The bromoalcohol was identified by NMR and chiral gas chromatography. ^1^H-NMR δ ppm 0.83 (d, *J* = 6.96 Hz, 3H) 1.15–1.33 (m, 7H) 1.34–1.44 (m, 1H) 1.45–1.55 (m, 2H) 2.05 (d, *J* = 4.64 Hz, 1H) 3.31 (dd, *J* = 10.21, 6.96 Hz, 1H) 3.48 (dd, *J* = 10.21, 3.25 Hz, 1H) 3.65–3.77 (m, 1H); ^13^C-NMR δ ppm 14.28 (2C, CH_3_) 22.79 (1C, CH_2_) 25.81 (1C, CH_2_) 29.37 (1C, CH_2_) 31.92 (1C, CH_2_) 35.35(1C, CH_2_) 40.94 (1C, CH_2_) 71.32 (1C, CH) [26]; chiral GC R_t_: 17.25 min.

## 4. Conclusions

An enzymatic tandem method was developed to prepare (*S*)-1-halo-2-octanols with >98% *ee* from racemic 1,2-octanediol. First, the synthesis of regioisomeric mixtures of halohydrin esters was carried out using described methods. The regioisomeric mixtures were then hydrolyzed selectively using both resting cells of *R. oryzae* and commercial lipases. The regioselectivity of these hydrolyses depended on the solvent and the biocatalyst used. Finally, Novozym^®^ 435 was used to carry out a kinetic resolution of the almost pure racemic mixture obtained previously. Using palmitic acid and the corresponding enzymes and ionic liquids, (*S*)-1-chloro-2-octanol and (*S*)-1-bromo-2-octanol were obtained with 26% and 15% overall yield, respectively, from 1,2-octanediol.

## Figures and Tables

**Table 1:**
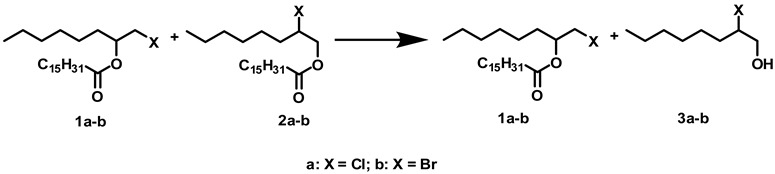
Percentage of hydrolysis of both palmitate regioisomers using various biocatalysts and solvents.

Entry	Biocatalyst	Solvent		
		*t*-Butanol	[OMIM][BF_4_]	[BMIM][PF_6_]
**1a**	*R. oryzae*	13		
**2a**	*R. oryzae*	100		
**1a**	Lipozyme	8	5	
**2a**	Lipozyme	100	89	
**1a**	Novozym	57	43	51*
**2a**	Novozym	100	85	53*
**1b**	*R. oryzae*	57		
**2b**	*R. oryzae*	100		
**1b**	Lipozyme	54	1	
**2b**	Lipozyme	100	90	
**1b**	Novozym	52	15	35*
**2b**	Novozym	100	91	52*

*3 h reaction.

**Table 2:**
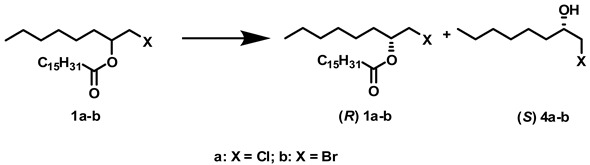
Percentage hydrolysis, *ee*_s_, and E values of 1-halo-2-octyl palmitates using various biocatalysts and solvents.

Entry	Biocatalyst	Solvent	% Hydrolysis	ee_s_^a^	E^a^
**1a**	Novozym	*t*-Butanol	57	90	40
**1a**	Novozym	[OMIM][BF_4_]	43	98	220
**1a**	Novozym	[BMIM][PF_6_]*	51	98	360
**1b**	*R. oryzae*	[OMIM][BF_4_]	57	47	4
**1b**	Lipozyme	*t*-Butanol	54	48	4
**1b**	Novozym	*t*-Butanol	52	90	50
**1b**	Novozym	[BMIM][PF_6_]*	35	> 99	> 340

* 3 h reaction; ^a^The enantiomeric excess of (*S*)-alcohol was determined by chiral GC, and from these figures, the E value was calculated according to Rakels *et al.* [28].
